# Array comparative genomic hybridisation (aCGH) analysis of premenopausal breast cancers from a nuclear fallout area and matched cases from Western New York

**DOI:** 10.1038/sj.bjc.6602784

**Published:** 2005-09-13

**Authors:** G Varma, R Varma, H Huang, A Pryshchepava, J Groth, D Fleming, N J Nowak, D McQuaid, J Conroy, M Mahoney, K Moysich, K L Falkner, J Geradts

**Affiliations:** 1 Department of Medicine, Roswell Park Cancer Institute, Buffalo, NY, USA; 2 Department of Biostatistics, Roswell Park Cancer Institute, Buffalo, NY, USA; 3 Department of Pathology and Laboratory Medicine, Roswell Park Cancer Institute, Buffalo, NY, USA; 4 Department of Cancer Genetics, Roswell Park Cancer Institute, Buffalo, NY, USA; 5 Department of Cancer Prevention and Epidemiology, Roswell Park Cancer Institute, Buffalo, NY, USA; 6 Department of Oral Biology, State University of New York, Buffalo, NY, USA

**Keywords:** amplification, array CGH, breast cancer, deletion, radiation

## Abstract

High-resolution array comparative genomic hybridisation (aCGH) analysis of DNA copy number aberrations (CNAs) was performed on breast carcinomas in premenopausal women from Western New York (WNY) and from Gomel, Belarus, an area exposed to fallout from the 1986 Chernobyl nuclear accident. Genomic DNA was isolated from 47 frozen tumour specimens from 42 patients and hybridised to arrays spotted with more than 3000 BAC clones. In all, 20 samples were from WNY and 27 were from Belarus. In total, 34 samples were primary tumours and 13 were lymph node metastases, including five matched pairs from Gomel. The average number of total CNAs per sample was 76 (range 35–134). We identified 152 CNAs (92 gains and 60 losses) occurring in more than 10% of the samples. The most common amplifications included gains at 8q13.2 (49%), at 1p21.1 (36%), and at 8q24.21 (36%). The most common deletions were at 1p36.22 (26%), at 17p13.2 (26%), and at 8p23.3 (23%). Belarussian tumours had more amplifications and fewer deletions than WNY breast cancers. HER2/*neu* negativity and younger age were also associated with a higher number of gains and fewer losses. In the five paired samples, we observed more discordant than concordant DNA changes. Unsupervised hierarchical cluster analysis revealed two distinct groups of tumours: one comprised predominantly of Belarussian carcinomas and the other largely consisting of WNY cases. In total, 50 CNAs occurred significantly more commonly in one cohort *vs* the other, and these included some candidate signature amplifications in the breast cancers in women exposed to significant radiation. In conclusion, our high-density aCGH study has revealed a large number of genetic aberrations in individual premenopausal breast cancer specimens, some of which had not been reported before. We identified a distinct CNA profile for carcinomas from a nuclear fallout area, suggesting a possible molecular fingerprint of radiation-associated breast cancer.

The incidence of breast cancer in young women is lower than in the postmenopausal age group. However, carcinomas in these patients are generally more aggressive and associated with poorer prognosis. The aetiology of premenopausal breast cancer is not clear. In a minority of patients, tumours develop on the basis of germline mutations in the BRCA1 and BRCA2 genes. Environmental exposure variables have been extensively studied as a causative factor of human mammary neoplasia. There is significant evidence, derived from diverse populations, that ionising radiation can cause breast cancer. Some examples are patients who received therapeutic radiation to the chest early in life for Hodgkin's disease (HD), patients who were treated with radiation for mastitis and other benign breast diseases, patients who received thymic irradiation, patients who underwent frequent fluoroscopies for pulmonary disease, and atomic bomb survivors (Behrens *et al*, 2000; Clemons *et al*, 2000; Gaffney *et al*, 2001; Land *et al*, 2003; Travis *et al*, 2003). Among these, the best-studied group are women who developed breast cancer after treatment for HD. It was reported that the risk of developing breast cancer was greatest if patients were treated under the age of 30, and hormonal stimulation of the irradiated breast tissue appeared to play an important role (Clemons *et al*, 2000; Travis *et al*, 2003). The risk clearly was dose dependent (Travis *et al*, 2003), and the median latency period was in the range of 15 years (Clemons *et al*, 2000). Similar observations held true for the Japanese atomic bomb survivors. Breast cancer risk was inversely related to age at exposure, there was a linear dose response, and the minimum latency period was 12 years (Land *et al*, 2003). Tumours arising in irradiated women may be associated with reduced overall survival (Gaffney *et al*, 2001).

Researchers at Roswell Park Cancer Institute (RPCI), including some of the authors of this paper, have been actively involved in several epidemiologic studies on a more recent group of probands, namely individuals exposed to radiation released by the 1986 Chernobyl nuclear accident (Mahoney *et al*, 2004). Although the reactor was located in Ukraine, neighbouring Belarus received about 70% of the radioactive fallout (Ermak *et al*, 2003). It is well documented that children exposed to that fallout have an increased cancer rate (Peterson *et al*, 1997). One of the most common radiation-induced malignancies is papillary carcinoma of the thyroid, and this tumour type was shown to harbour molecular abnormalities that differed from those in sporadic thyroid cancers (Tuttle and Becker, 2000; Ermak *et al*, 2003). There also was an increase in breast cancer incidence in parts of Belarus after the Chernobyl accident, especially in rural areas and in premenopausal women (Sasnouskaya and Okeanov, 2000).

In theory, breast carcinomas associated with ionising radiation should facilitate insight into the molecular pathogenesis of early-onset breast cancer, yet few such studies have been published. In one paper, post-HD breast carcinomas were found to have more microsatellite alterations compared to sporadic tumours (Behrens *et al*, 2000). In another group of post-HD breast cancer patients, there were no significant differences compared with sporadic cases with regard to BRCA1, BRCA2, oestrogen receptor (ER), PR, HER2 and p53 status (Gaffney *et al*, 2001). Several *in vitro* studies are of relevance. It was shown that human mammary epithelial cells (HMEC) in culture can be transformed by *γ*-irradiation (Wazer *et al*, 1994). Subsequent studies showed that radiation induced nonrandom chromosomal changes in HMEC (Durante *et al*, 1996; Yang *et al*, 1997). Finally, irradiation of nontransformed MCF-10F cells led to altered expression of 49 of 190 genes assayed (Roy *et al*, 2001).

Comparative genomic hybridisation (CGH) is one tool to investigate the molecular pathology of human tumours. Conventional CGH is based on competitive *in situ* hybridisation of normal metaphase spreads by two differentially labelled whole genomic DNAs, one derived from tumour tissue and the other from a normal reference. Regions of altered DNA copy number (losses and gains) in the tumour are quantitated as ratio changes along metaphase chromosomes. The resolution of this technique is in the range of 10–20 Mb. In this study, we have used array CGH to obtain better resolution to facilitate identification of novel candidate breast cancer genes. High-density bacterial artificial chromosome (BAC)-based arrays developed at RPCI were employed to screen for DNA copy number gains and losses in premenopausal breast cancers from two geographically distinct areas in an attempt to identify genetic changes that may be specific to early-onset tumours and/or radiation exposure.

## MATERIALS AND METHODS

### Tissues

In all, 55 frozen samples of breast cancers were obtained from premenopausal patients from Western New York (WNY) and Belarus. Samples from Belarus were collected and snap frozen between August 2002 and January 2003. The tumours were collected from women who resided in the Gomel area since April 1986. They were transported on dry ice to the United States and processed at RPCI. The WNY samples were obtained through the tissue procurement facility at RPCI. Patients with a strong family history of breast cancer and cases with known BRCA1/2 mutations were excluded. This study was approved by the RPCI Institutional Review Board. All samples were evaluated morphologically and only those with more than 50% tumour cellularity were included. Preliminary experiments had shown that >30% tumour cellularity was sufficient. Duplicate assays and dye swapping experiments were performed for a subset of samples, showing very good reproducibility. Genomic DNA was extracted from all samples using TriZol (Invitrogen, Grand Island, NY, USA). In total, 47 samples met the quality criteria and were included in the final analysis. Patient characteristics are shown in Table 1. All Gomel patients were Caucasian, as were the great majority of RPCI patients (except for two African American women). The age of the patients ranged from 24 to 50 years. There were 34 primary tumours and 13 lymph node metastases. This cohort included five paired cases from Belarus.

### Her2 protein expression assays

The immunohistochemical assay for Her2 expression in formalin-fixed, paraffin-embedded tumour sections followed the Herceptest protocol (Dako, Carpinteria, CA, USA), and the stains were scored from 0 and 1+ (negative) to 2+ and 3+ (positive), using recommended guidelines. For Western blotting, 50 *μ*g of protein was used per sample (extraction from tissue utilized the TriZol protocol). Protein was resolved over 8% SDS–PAGE and transferred to a PVDF membrane. The blot was blocked in blocking buffer (5% nonfat dry milk, 10 mM Tris (pH 7.5), 10 mM NaCl, and 0.1% Tween-20) for 1 h at room temperature. The membrane was then incubated with the Herceptest antibody (Dako) at a dilution of 1 : 500 at 4°C overnight. This was followed by incubation with a goat anti-mouse horseradish peroxidase-conjugated secondary antibody (KPL, Gaithersburg, MD, USA) at a dilution of 1 : 5000 at room temperature for 1 h. Protein bands were visualised by the SuperSignal West Pico Chemiluminescent Substrate kit obtained from PIERCE (Rockford, IL, USA) and exposed with Kodak X-Ray film.

### Array-based CGH (aCGH)

**Figure 1:**
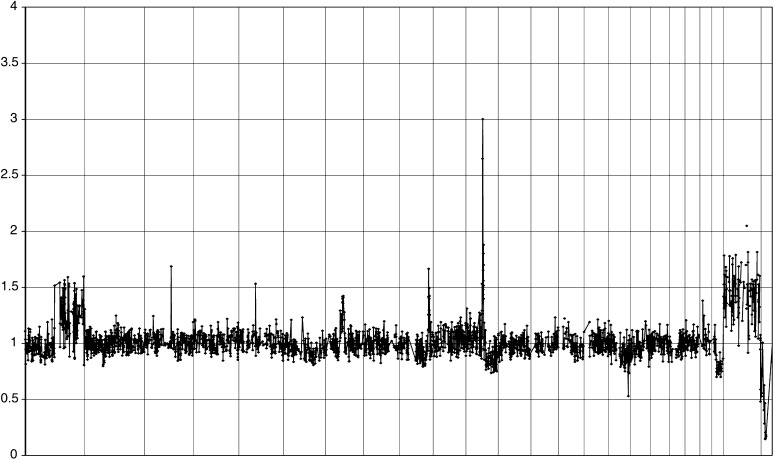
aCGH of a representative tumour. DNA from a primary breast cancer was hybridised with normal male reference DNA. The whole genome is arranged along the *x*-axis from left (1p) to right (X, Y). The chromosomal boundaries are indicated by vertical lines. The *y*-axis is linear. A number of distinct amplifications (e.g. 3q, 9q, 11) and deletions (16q) as well as larger regions of DNA copy gains (e.g. 1q) and losses (11q, 22q) are easily recognised.

The CGH arrays were prepared in the Microarray Core Facility at RPCI according to established protocols (Snijders *et al*, 2001; Cowell and Nowak, 2003; Cowell *et al*, 2004a). A total of 3084 BAC clones were common to all arrays used in this series of experiments (average resolution ∼1 Mb). The WNY and Belarussian samples were coded and then assayed concurrently and blindly. Genomic DNA (0.5 *μ*g) was fluorescently labelled by random priming in a 100 *μ*l reaction containing the DNA, random primers solution, appropriate buffers and Cy3- or Cy5-dCTP-labelled nucleotides. Labelling occurred with the addition of appropriate agents and incubation overnight at 37°C. Arrays were hybridised with appropriate solutions for 16 h. Slides were washed, dried and immediately scanned using an Affymetrix 428 scanner (Cowell *et al*, 2004b). Image analysis was performed using the ImaGene version 4.1 software from BioDiscovery Inc. The reference was pooled normal male DNA. Output of the image analysis was processed by an in-house Perl program to calculate log-transformed and normalised mean ratios of test to reference fluorescence intensities. Any BAC that had less than two replicates flagged as good or a standard error greater than 0.15 was excluded. Map positions were identified by querying the human genome sequence (July 2003 Build) at http://genome.ucsc.edu. A sample output showing intensity ratios across the whole genome for an individual tumour is shown in Figure 1.

### Data analysis

Copy number aberrations (CNAs) were determined at the clonal level for each individual coded sample in a blinded manner. The genome-wide mean and standard deviation of the log ratios were calculated for the autosomal chromosomes and the X chromosome separately. Thresholds for amplification and deletion were set at two standard deviations from the mean in both directions. Contiguous regions of amplification or deletion were identified by flagging clones on the array based on adjacent chromosomal locations. Recurrent amplifications and deletions were determined by the frequency of these events among all samples or within specific groups.

Unsupervised hierarchical clustering was performed using the TIGR Multi-experiment Viewer (MeV version 2.2) software (Saeed *et al*, 2003). Only clones that were included in at least 45 of 47 samples were considered. A filtered set of 202 clones with a high variability across all samples (standard deviation/mean>0.3) was used for the hierarchical clustering. Clusters were generated using the average linkage method with Pearson's correlation coefficient as the similarity metric.

Association between groups was tested from contingency tables using the χ^2^ Pearson statistic. Resulting *P*-values are shown as appropriate.

## RESULTS

In all, 47 samples that met all quality control criteria including tumour cellularity and patient age were included in the final analysis. Patient charactertistics are shown in Table 1. The two groups from WNY and Gomel were well matched for ethnic background, age, extent of disease (tumour size, nodal involvement, stage), tumour histology, grade and ER status. The average number of CNAs in the breast carcinomas was 76 (37 gains and 39 losses). Tumours from WNY, older patients, and HER2-positive cancers had more deletions and fewer amplifications than tumours from Belarus, younger patients, and HER2-negative cancers, respectively (Table 2). Primary tumours and lymph node metastases, smaller and larger tumours, ER+ and ER− cases, and carcinomas with and without associated nodal metastases had comparable frequencies of copy number gains and losses.

**Figure 2:**
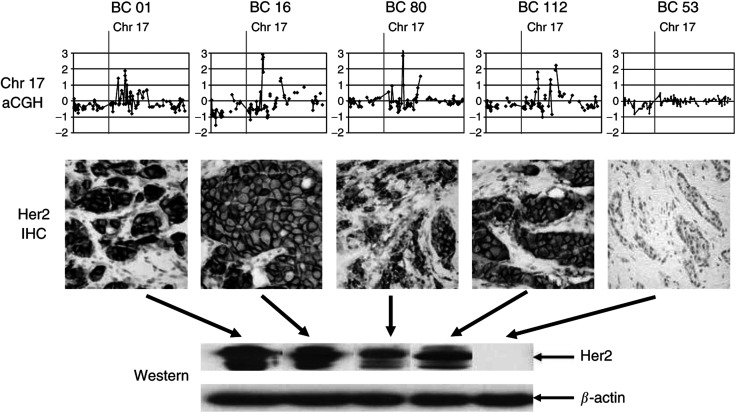
HER2 amplification and overexpression. Nine tumours from eight patients showed amplification at 17q21. The chromosome 17 aCGH profiles of four of them are shown at the top along with one nonamplified sample (the *y*-axis is on a log_2_ scale). This was associated with Her2 protein overexpression by immunohistochemistry (middle) and Western blot analysis (bottom).

A representative diagram of genome-wide amplifications and deletions is shown in Figure 1. Most breast cancers displayed both distinct single BAC amplifications and deletions as well as broader areas of DNA gains and losses. A total of 152 CNAs occurred in more than 10% of samples, including 92 copy number gains and 60 losses. The majority of DNA gains (72%) were on the long arms of chromosomes 1 and 8. Most of them were gains of one or two copies, but a subset was higher level amplifications (three copies or more, Table 3). The most common amplifications were at 8q13.2 (49%), at 1p21.1 (36%), and at 8q24.21 (36%). Gains at loci containing the well-known breast oncogenes c-myc (at 8q24.21), HER2 (at 17q21.1), and cyclin D1 (at11q13.3) were also frequent (26, 19, and 13%, respectively) (Table 3). In an initial validation experiment, HER2 amplification detected by array CGH was correlated with protein expression using immunohistochemistry and Western blot analysis (Figure 2). Amplified tumours showed high levels of the Her2 oncoprotein, while nonamplified cases were negative. While the majority of chromosomal gains had previously been described, we identified seven novel recurrent gains (as indicated in Table 3), five of which were in areas on 8q that had not been reported to be common amplification sites in breast cancer.

The largest number of DNA losses was on chromosome 17. In most instances, one allele was lost, but possible homozygous deletions were also observed (Table 4). The most common deletions were at 1p36.22 (26%), at 17p13.2 (26%), and at 8p23.3 (23%). We identified nine novel recurrent losses, seven of which were on chromosome 22 (Table 4).

In the five paired samples, some genetic changes were common to the primary tumour and the lymph node metastasis. The number of shared changes varied markedly from case to case. However, all cases were characterised by a large number of discordant CNAs. Metastatic tumours consistently developed more gains than losses (Table 5).

**Figure 3:**
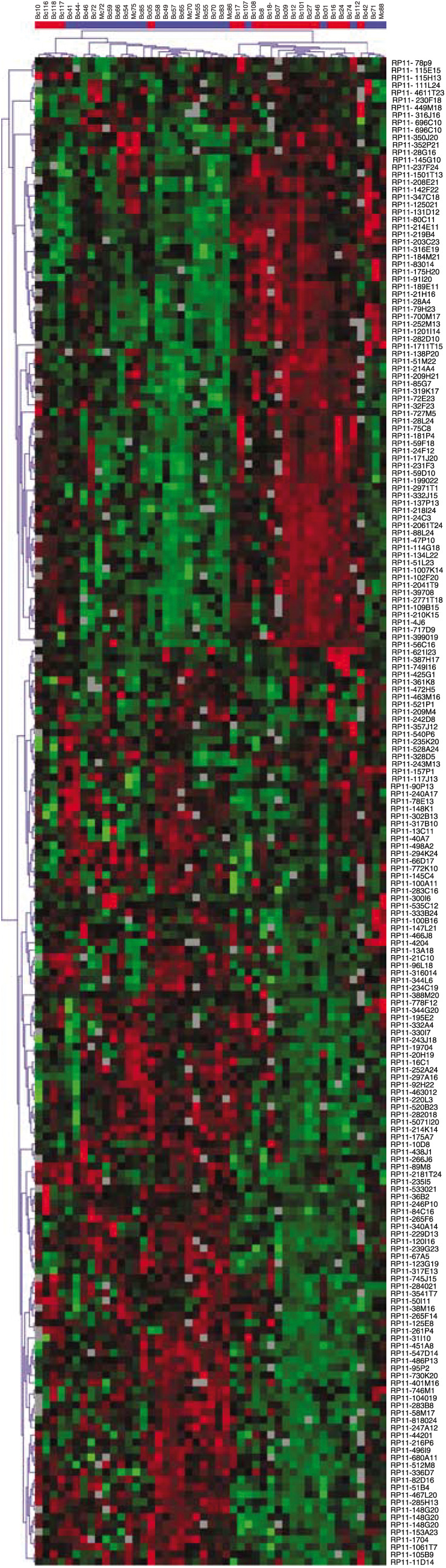
Cluster analysis (dendrogram). Unsupervised hierarchical clustering based on 202 BAC clones (vertical) yielded two main arms (horizontal): 26 tumours predominantly from Belarus (blue) on the left and 21 tumours mainly from WNY (red) on the right.

Unsupervised hierarchical clustering using 202 discriminating BAC clones produced a dendrogram with two distinct arms: one predominantly composed of WNY carcinomas and the other mostly comprised of Belarussian samples (*P*<0.001) (Figure 3). These two arms were not significantly different with regard to patient age, primary tumours *vs* metastases, tumour size, nodal status, stage, grade, ER, or HER2 status. A total of 50 BAC clones were differentially amplified or deleted in premenopausal breast cancers from WNY and Belarus, and 25 of these contained named genes (Table 6). Of particular interest were 10 BAC clones that were amplified selectively in Belarussian tumours. Moreover, three of these BACs were specifically deleted in WNY cases. Two BAC clones with known genes were significantly more often amplified, and 13 were more frequently deleted in WNY tumours.

## DISCUSSION

There is a paucity of data on genomic changes in early-onset breast cancer. The study reported here benefitted from a unique population, namely premenopausal women from Belarus who were exposed to significant fallout radiation from the 1986 Chernobyl nuclear accident. It has been suggested that Chernobyl-related cancers may be the best model to study the carcinogenic effect of low-dose radiation (Ermak *et al*, 2003). We were able to compare the genomic abnormalities in the Belarussian cancers to those in similar tumours from a nonexposed area that were well matched for ethnic background, age, tumour characteristics, and extent of disease.

By utilising high-density aCGH technology with the capacity to screen for DNA copy changes across the entire genome at high resolution, we identified a larger number of chromosomal abnormalities than previous breast cancer studies. Links to genomic databases helped to identify candidate genes for further investigation. A small number of published studies applied aCGH technology to breast cancer cells, but only to a very limited extent (Pinkel *et al*, 1998; Albertson *et al*, 2000; Kauraniemi *et al*, 2001; Hyman *et al*, 2002; Pollack *et al*, 2002; Lage *et al*, 2003). Almost all of these utilised a significantly smaller number of specimens. Some of them focused only on cell lines (Hyman *et al*, 2002; Lage *et al*, 2003) or on individual chromosomes (Pinkel *et al*, 1998; Albertson *et al*, 2000; Kauraniemi *et al*, 2001). We are not aware of any published reports on array or conventional CGH analysis of DNA abnormalities in irradiated cells. Likewise, our study may be the first to screen for somatic genetic changes specifically in premenopausal tumours, providing new insight into the molecular pathology of early-onset breast cancer. The average number of total CNAs in our cohort of tumours was 76 (range 35–134), which is approximately eight times the average number of CNAs in breast cancers detected by conventional CGH (Isola *et al*, 1995; Kuukasjärvi *et al*, 1997; Aubele *et al*, 2000a, 2000b; Zudaire *et al*, 2002), demonstrating the superior sensitivity of our assay. This is in keeping with the density of the BAC arrays used (some 3000 elements, average resolution ∼1 Mb).

Several groups have used conventional CGH to probe for genetic aberrations in invasive breast carcinomas. Tirkkonen *et al* (1998) found that the pattern of chromosomal gains and losses depended on tumour size, grade, and receptor status. They described gain on 1q as an early event and gain on 8q as a marker for advanced breast cancers. We have identified similar recurrent gains on 1q and 8q, which were the most commonly affected chromosomal arms in our series (Table 3). In a study of T2 (>2 cm) node-negative breast cancers, a high overall number of genetic aberrations was correlated with poor prognosis, and an increased copy number at 8q and 20q13 indicated an aggressive phenotype (Isola *et al*, 1995). Another similar study of invasive ductal carcinomas found that gains on 1q, 11q, 17q, and 20q were associated with poor prognosis (Zudaire *et al*, 2002). These DNA changes were common in our series as well, and this observation is in agreement with the more aggressive clinical course of premenopausal breast cancer. Nine of 47 tumours showed amplification of a BAC clone including HER2. This copy gain rate (19%) is in keeping with numerous previous studies on HER2 amplification in breast cancer and proved to be among the most common abnormalities in our study. Additional commonly amplified BACs included the well-known breast cancer oncogenes c-myc (at 8q24.21) and cyclin D1 (at 11q13.3), further demonstrating adequate sensitivity of our method. Novel recurrent CNAs with potential target genes included gains at 3q25.31, 6q26, 7q36.3, 13q32.2, and 16p11.2 (Table 3). Seven recurrent amplification loci, most of which were found on 8q, had not been reported to be frequent events in breast carcinomas.

Recurrent deletions were less common, and no single event was found in more than 26% of the samples. Losses were most frequently observed on chromosome 17, which is in keeping with published cytogenetic data. However, nine of the remaining recurrent deletions, including seven on chromosome 22, had not been reported before (Table 4). Potential target genes included KREMEN1 (on 22q12.1), which is a component of the Wnt signalling pathway (Mao *et al*, 2002).

It is conceivable that the novel chromosomal gains and losses described here may be the hallmark of premenopausal breast cancer. While our high-density BAC arrays appeared to have adequate sensitivity, an initial validation experiment suggested that the detected abnormalities may also be specific and verifiable by alternate techniques. The tumours with amplification of the HER2 locus also showed overexpression of the Her2 oncoprotein, while the nonamplified tumours showed no immunoreactivity (Figure 2).

Our series included five matched pairs of primary tumours and nodal metastases from Belarus. As expected, a number of chromosomal abnormalities were common to both the metastasis and the parental tumour, although the number of shared CNAs varied significantly from case to case (Table 5). Importantly, all cases were characterised by a large number of discordant events. Primary tumours developed a smaller, larger, or similar number of CNAs compared to the secondary lesions. They tended to have more deletions than losses. In contrast, nodal metastases consistently were marked by a larger number of amplifications over deletions. These findings suggest that metastases may form at variable points in the evolution of a breast cancer, and that primary tumour and metastasis independently develop additional genetic changes. This adds to the growing body of evidence that metastatic breast cancers may be biologically distinct from their parental tumours.

As detailed in Table 2, the genetic changes were not evenly distributed among the premenopausal breast cancers. While chromosomal gains and losses were not dependent on tumour site or size, nodal involvement, or ER status, younger age and HER2 negativity were associated with a smaller number of deletions and more amplifications. Likewise, tumours from Belarus had more DNA gains and fewer deletions than carcinomas from WNY. One of the most interesting observations was that breast cancers from Gomel had a DNA profile that was distinct from that of an age- and stage-matched control group treated at RPCI. Strikingly, when all 47 cases were subjected to unsupervised hierarchical clustering, two distinct groups emerged: one mostly comprised of Belarussian breast cancers and one mainly comprised of cases from WNY (Figure 3). This segregation was statistically highly significant (*P*<0.001). All of the other variables (age, extent of disease, grade, receptor status) were similarly distributed in the two arms. In all, 50 BAC clones were differentially amplified or deleted in the two groups of tumours, and half of these contained named genes. In total, 10 chromosomal gains were specific to the Belarussian samples, and it is tempting to speculate that these may represent the molecular hallmark of radiation associated breast cancer. Potential target genes included the MDM2-related gene MDM4 (at 1q32.1) and SULT1A3 (at 16p11.2) encoding an enzyme involved in the metabolism of catecholamines and related compounds (Hildebrandt *et al*, 2004). WNY tumours were characterised by a significantly higher number of deleted BAC clones. It is unclear whether the chromosomal abnormalities in the tumours from Gomel are important in their pathogenesis or merely markers of genomic susceptibility to radiation damage. There are no associated epidemiologic or dosimetry data for the individual samples from Belarus so that, at this juncture, we cannot be sure that the specific changes in the tumour DNA are in fact due to radiation exposure. While the Belarussian population is ethnically homogeneous (Ermak *et al*, 2003), and while the racial background of the breast cancer patients from the Gomel area was similar to that in the WNY cohort, we cannot rule out that other endogenous or exposure variables such as smoking or diet may account for the difference in genomic abnormalities, although there is no evidence that such environmental factors impact on the pattern of chromosomal aberrations in breast carcinomas.

In conclusion, our study significantly adds to the existing body of knowledge by (a) detailing a number of previously undescribed recurrent chromosomal gains and losses in premenopausal breast cancers; (b) focusing on a unique cohort of breast carcinomas associated with prolonged low-dose radiation exposure; and (c) describing a set of CNAs that are specific to tumours from a nuclear fallout area. Our findings may provide the basis for future studies on the molecular pathogenesis of early-onset breast cancer.

## Figures and Tables

**Table 1:** Patient characteristics

	**WNY**	**Belarus**	***P*-value**
Age (median, years)	41.5	45.5	
⩽43	12	10	0.23
>43	8	12	
			
Caucasian	18	22	
African American	2	0	0.43
			
*Histology*			
Ductal	17	20	
Lobular	2	0	0.29
Other/unknown	1	2	
			
*Grade*			
1–2	7	5	
3	13	14	0.81
Unknown	0	3	
			
*Size*			
T1	4	8	
T2–T4	16	12	0.30
Unknown	0	2	
			
*LN status*			
Negative	6	5	0.85
Positive	14	17	
			
*Stage*			
I	2	4	
II	11	12	0.71
III	7	6	

WNY=Western New York; LN=lymph node.

**Table 2:** Distribution of CNAs

	**Average gains**	**Average losses**	**Average total CNAs**
*WNY*	32^a^	44	76
Belarus	42^a^	35	77
			
Age under median (⩽43)	43^b^	33^c^	76
Age over median (>43)	33^b^	45^c^	78
			
Small tumours (T1)	39	36	75
Large tumours (T2–T4)	37	40	77
			
Lymph node negative	39	37	76
Lymph node positive	35	41	76
			
Primary tumours	37	41	78
Lymph node metastases	40	33	73
			
ER positive	31	49	80
ER negative	37	37	74
			
HER2 negative	41^d^	35	76
HER2 positive	28^d^	46	74
			
Total	37	39	76

CNA=copy number aberrations; WNY=Western New York.

^a^*P*=0.034.

^b^*P*=0.039.

^c^*P*=0.054.

^d^*P*=0.037.

**Table 3:**
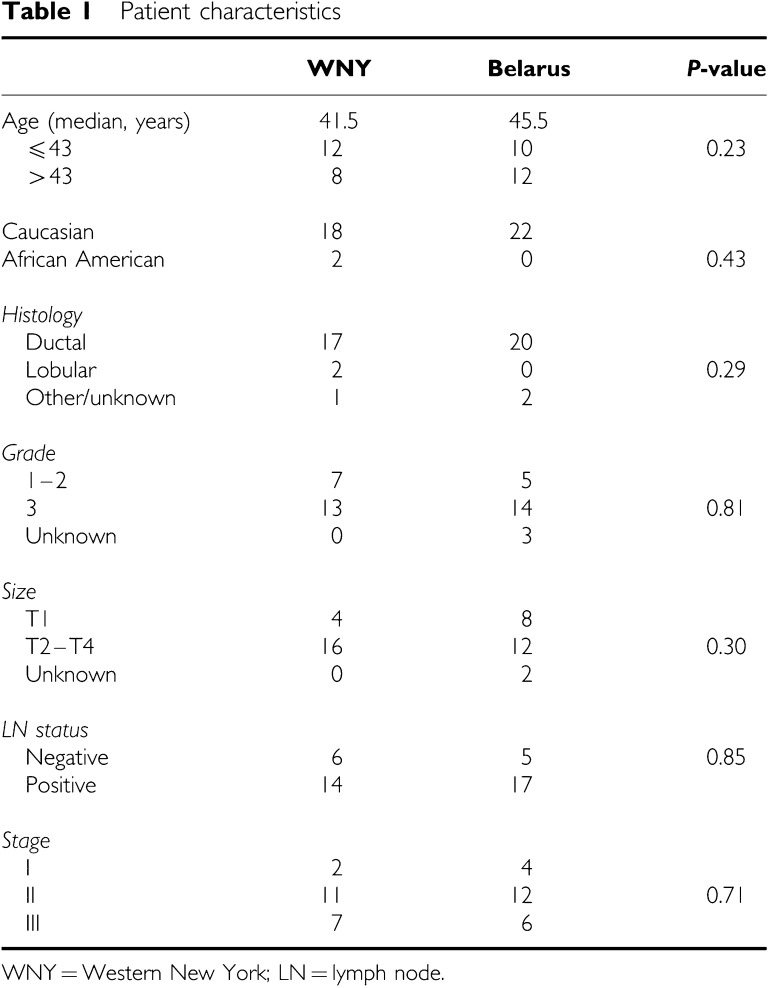
Recurrent gains arranged by chromosomal location (*n*=92)

**Table 4:**
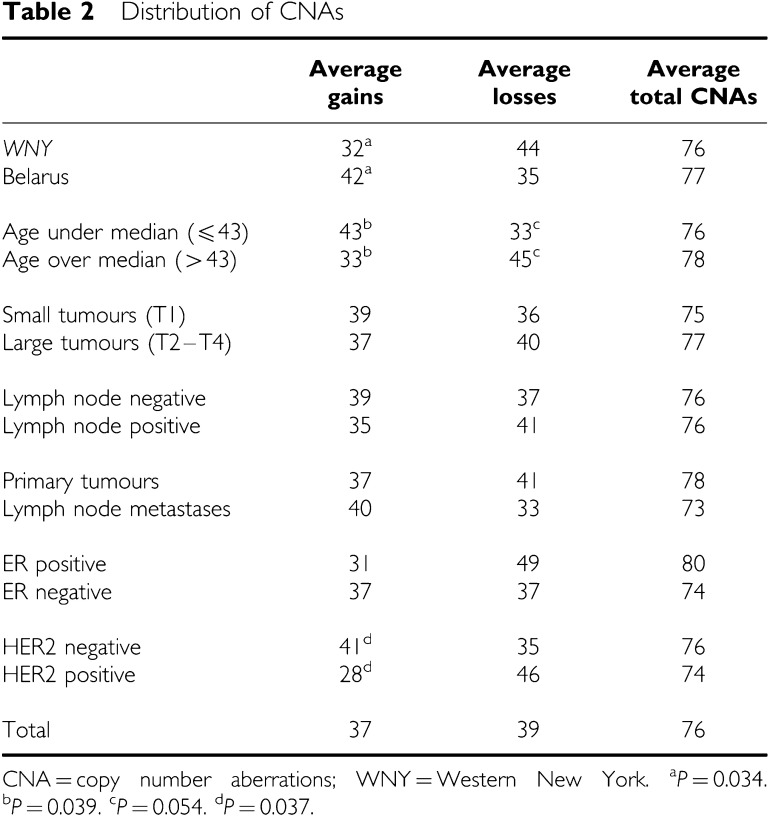
Recurrent losses arranged by chromosomal location (*n*=60)

**Table 5:** Copy number gains and losses in paired primary breast cancers and their nodal metastases

	**Genetic changes common to both primary and metastasis**		**Discordant genetic changes**			
			**In primary only**		**In nodal metastasis only**	
**Paired case #**	**Gains**	**Losses**	**Gains**	**Losses**	**Gains**	**Losses**
1	63 (33)	9 (7)	16 (11)	9 (8)	30 (25)	12 (11)
2	19 (13)	10 (7)	21 (15)	26 (25)	31 (30)	15 (13)
3	20 (11)	1 (1)	48 (38)	37 (30)	42 (35)	22 (20)
4	7 (5)	9 (5)	25 (20)	34 (31)	36 (33)	24 (23)
5	13 (7)	0 (0)	13 (12)	91 (63)	42 (29)	4 (3)

Number of amplified or deleted BAC clones (number of affected chromosomal bands in parentheses).

**Table 6:** Genetic changes distinguishing premenopausal breast cancers from WNY and Gomel, Belarus

			**WNY**		**Belarus**	
**BAC clone (RP11-)**	**Chr. loc.**	**Candidate genes**	**Ampl.**	**Del.**	**Ampl.**	**Del.**
425I11	8q21.3	DECR1, C8orf1	9		2	
122C21	8q21.3	CBFA2T1	6		2	
157P1	20q13.33	OSBPL2, ADRM1, LAMAS	0		**8**	
148K15	1q32.1	MDM4	0		**6**	
461N23	13q32.3	EBI2, GPR18	0		**6**	
150L7	1q32.1	PKP1	0		**5**	
243M13	1q32.1	CNTN2, RBBP5, ABO18299	0		**5**	
455F5	16p11.2	SULT1A3, ALDOA, TBX6, CORO1A, MGC5178	0		**5**	
465L10	20q13.12	PLTP, ZNF335, SLC12A5, NCOA5	0		**5**	
252A24	16q22.3	PSMD7, GLG1	0	6	**3**	1
2I16	5q35.3	COL23A1	1	4	**5**	0
58M17	16p13.2	USP7	0	3	**4**	0
73F15	17q11.2	CRLF3		6		0
126L15	7q22.1	ZAN, EPHB4, ACHE		3		0
211E17	11p15.4	TRIM3, ILK, TAF10, CLN2, PCDH16		3		0
35J17	19q13.42	ZNF331		3		0
571M6	12q14.1	CDK4		3		0
746M1	17p11.2	USP22, DKFZp5660084, C17orf35		3		0
36J16	17q21.32	NDP52, HOXB13		8		2
208J12	17p13.2	TRV1, CARKL, CTNS		8		2
61B16	17p13.3	CT120, GEMIN4		8		3
213L15	22q12.1	KREMEN1		6		1
2J15	19q13.32	CALM 3, PTIGR, GNG8		6		1
89M8	8p21.2	RHOBTBN2, TNFRSF10B		6		1
298C17	19p13.2	EIF3S4, DNMT1, P2RY11		6		1

WNY=Western New York; Chr. loc.=chromosomal location; Ampl.=amplification; Del.=deletion.

In all, 25 (out of 50) distinguishing BAC clones contained named genes (listed).

Ten BAC clones (bold) were selectively amplified in Belarussian breast cancers and may represent signature events related to radiation exposure.
